# High-efficiency generation of far-field spin-polarized wavefronts via designer surface wave metasurfaces

**DOI:** 10.1515/nanoph-2022-0006

**Published:** 2022-03-15

**Authors:** Weikang Pan, Zhuo Wang, Yizhen Chen, Shiqing Li, Xiaoying Zheng, Xinzhang Tian, Cong Chen, Nianxi Xu, Qiong He, Lei Zhou, Shulin Sun

**Affiliations:** Shanghai Engineering Research Center of Ultra-Precision Optical Manufacturing, Department of Optical Science and Engineering, School of Information Science and Technology, Fudan University, Shanghai 200433, China; Yiwu Research Institute of Fudan University, Chengbei Road, Yiwu City, 322000 Zhejiang, China; State Key Laboratory of Surface Physics and Key Laboratory of Micro and Nano Photonic Structures (Ministry of Education), Fudan University, Shanghai 200433, China; School of Electronic Information, Wuhan University, Wuhan 430072, China; Changchun Institute of Optics, Fine Mechanics and Physics, Chinese Academy of Sciences, Changchun, Jilin 130033, China; Collaborative Innovation Center of Advanced Microstructures, Nanjing 210093, China

**Keywords:** far-field wavefront, metasurface, Pancharatnam–Berry phase, propagation wave, surface wave

## Abstract

Achieving a pre-designed scattering pattern from an ultra-compact platform is highly desired for on-chip integration optics, but conventional techniques suffer from the limitations of bulky size, wavelength-scale modulation and low efficiency. Here, we propose a new strategy to *efficiently* generate arbitrary *spin-polarized* scattering far-field patterns from surface-wave (SW) excitations on a designer Pancharatnam–Berry (PB) metasurface. We find that a PB meta-atom serves as a subwavelength scatter to decouple impinging SW to a *spin-polarized* propagating wave (PW) with tailored amplitude and phase, and thus interference among PWs generated by scatterings at different PB meta-atoms can generate a tailored far-field pattern. As a proof of concept, we design and fabricate a series of PB metasurfaces in the microwave regime and experimentally demonstrate that they can generate desired radiation patterns within a broad frequency band, including unidirectional radiation, line/point focusing, vortex beam and hologram. These findings may stimulate important applications in on-chip integrated photonics.

## 1 Introduction

Surface waves (SWs) are eigen electromagnetic (EM) modes bounded at dielectric/metal surfaces [1], [2], [3]. Due to subwavelength resolution and local-field enhancement characteristics, SWs can find numerous applications in integration-optics, such as sub-diffraction imaging [4, 5], sensing [6, 7], plasmonic laser [8], [9], [10], [11], on-chip plasmonic circuit [12], [13], [14], [15], and enhanced nonlinear optics [16, 17]. In these applications, a crucial issue is to find appropriate devices to efficiently couple impinging propagating waves (PWs) into SWs inside the on-chip devices. Conventional PW–SW couplers, such as prism couplers [18, 19], grating couplers [20] and fibers coupler [21, 22], are bulky and less efficient. Recently, gradient metasurfaces exhibiting reflection/transmission phases linearly varying in space were found as efficient PW–SW couplers [23]. Different types of meta-couplers were proposed and experimentally characterized, working in reflection [24], [25], [26], [27] or transmission mode [28, 29], under excitations of PWs with linear or circular polarizations. Integrated with active elements, the metasurfaces can further exhibit tunable or reprogrammable functionality to manipulate impinging waves [30, 31].

On the other hand, achieving pre-designed far-field radiations from SWs flowing on a planar device have attracted much attention from science and technology recently. Scatterings of SWs by small objects/apertures usually generate significant beam divergence [10]. To address this issue, Lezec et al. added periodic textures surrounding a small aperture in a metal film to help collimate light beam emerged from the aperture, based on Bragg interferences of waves emitted through SW scatterings at these periodic structures [32]. Such a strategy was further applied to realize edge-emitting-semiconductor lasers with small angular divergence [33, 34]. Utilizing such a scheme, more fascinating beam-control effects were demonstrated with SW excitations on flat devices, such as far-field focusing [35], [36], [37], Airy beam [38, 39] and vortex beam [39], and holograms [40], [41], [42], [43]. Such an approach can find numerous promising applications in future on-chip optics, such as planar antenna [44, 45], mini-projector [46], [47], [48], and virtual reality displays [49].

**Figure 1:**
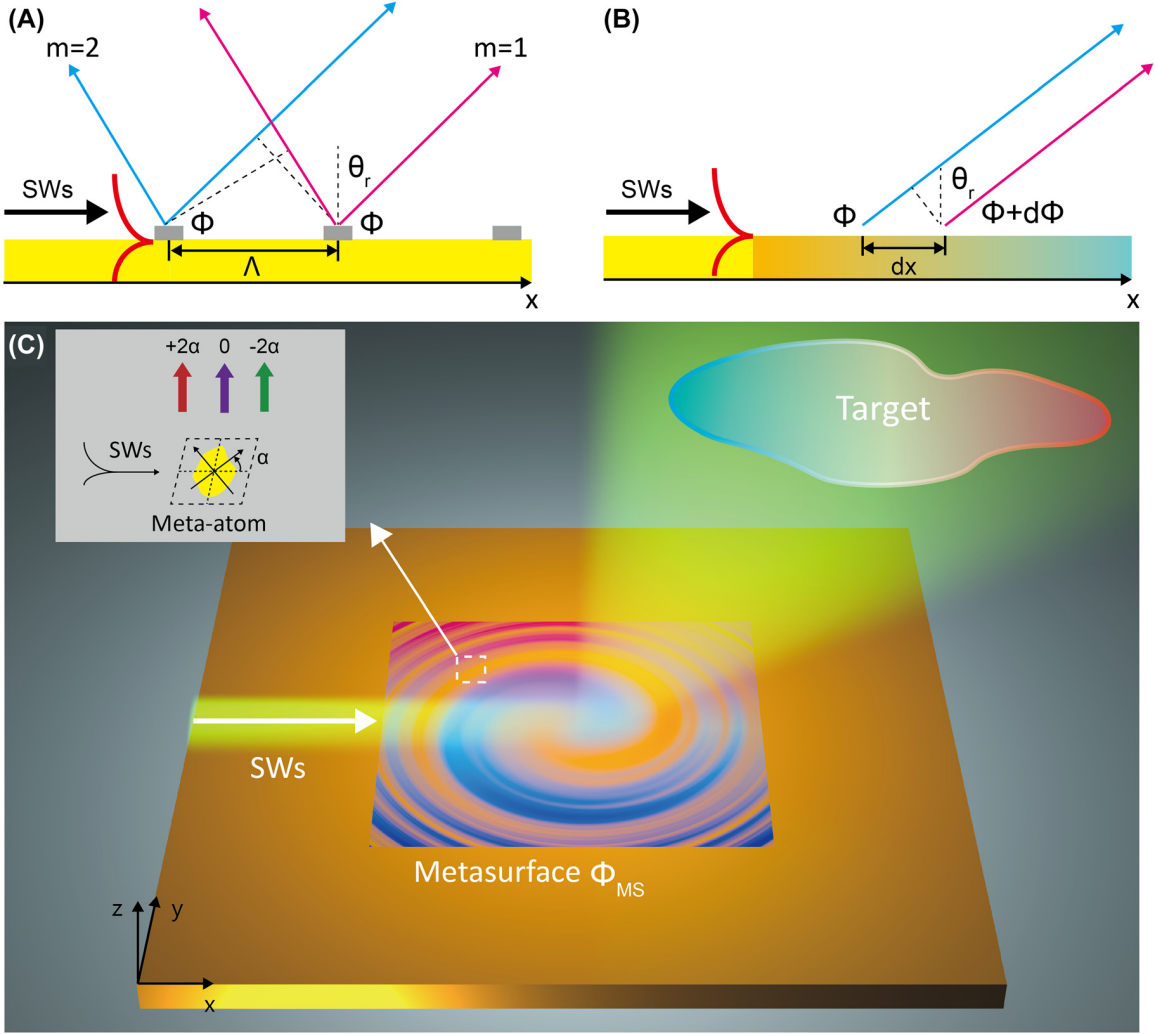
Schematics to illustrate physical concept of SW–PW radiations. (A) Physical concept of SW–PW radiations with Bragg structure. Based on Bragg scattering, the SWs are decoupled into different PW modes with the blue and red lines representing two out-going optical paths emitted by adjacent structures. (B) Physical concept of SW–PW radiations via the phase gradient provided by gradient metasurface. (C) Arbitrary scattering far-field patterns enabled by SWs decoupling with gradient metasurface. As shown in the inset, three different scattering mode are excited inside the meta-atom (of an orientation angle α ), including the LP normal mode without geometric phase, LCP and RCP anomalous modes with the radiation phase of ±2α . The interference of the scattering fields from different sub-cells form the desired radiation pattern.

However, these Bragg-type devices suffer from several limitations. Taking the Bragg grating as an example (see Figure 1A), while scatterings of an SW flowing across such a device can form a linearly polarized main beam in the far-field due to constructive interference, high-order beams may also be formed which degrades the performance of the device (see numerical demonstrations in Section A of Supplementary Material). The key issue is that Bragg devices can only control the scattered waves at the wavelength scale, but cannot control the amplitude, phase and polarization of wave scatterings at the deep-subwavelength scale, which significantly limit their abilities to freely engineer the far-field radiations.

In this paper, we propose a new strategy to achieve arbitrary *spin-polarized* far-field scattering patterns based on metasurfaces. Injecting an SW to excite a carefully designed PB meta-device (see Figure 1C), each meta-atom inside the device can serve as a subwavelength scatter to convert incident SW to circularly polarized PW with amplitude and phase determined by the structure and orientation of local meta-atom. Interferences among those scattered waves can form a desired far-field wavefront. We use this strategy to design/fabricate a series of microwave meta-devices and experimentally demonstrate various far-field scattering patterns, including unidirectional emission, focusing, vortex beam, and hologram. Compared to Bragg-type devices, our strategy exhibits the merits of single-mode operation, high-efficiency, high-resolution and being able to realize spin-polarized beams.

## 2 Physical concept and meta-atom design

Consider that an impinging SW with transvers-magnetic (TM) polarization travels across a carefully designed PB metasurface (see Figure 1B). As the impinging SW hits a PB meta-atom, electromagnetic resonances of meta-atom can be excited. Assuming that the meta-atom is deep-subwavelength in size and neglecting the spatial variation of $E_{\left|\right|}$ with the space occupied by the meta-atom, we can approximately view the meta-atom being excited by a time-oscillating homogeneous ${\bar{E}}_{\left|\right|}$ field [50]. Employing the same analysis as in Ref. [51, 52], we find that three different types of scattered waves can be generated, which are a linearly polarized normal mode carrying no additional phase and two circularly polarized abnormal modes carrying opposite PB phases, Φ=±2α , with sign determined by the chirality of the radiation beams. Here, α is the orientation angle of the meta-atom, and the strength of these scattered waves are related to the polarization conversion ratio (PCR) of the meta-atom [52], [53], [54]. Interferences of waves scattered by different meta-atoms can thus form three beams. While that corresponding to the normal mode forms an SW flowing forward, that corresponding to the abnormal mode exhibiting one particular chirality can form the desired target far-field radiation. Finally, interferences of abnormally scattered waves exhibiting another chirality are out of interest in this article, since they usually do not form any meaningful far-field pattern (see more discussions in Section B of Supplementary Material).

As a particular example, we illustrate how to design the PB meta-device aiming to realize the directional far-field radiation (see Figure 1B) with left circular polarization (LCP). To determine the $\Phi \left(\vec{r}\right)$ distribution of the PB meta-device for generating a far-field radiation beam along an angle $\theta_{r}$ , optical path analysis (see Figure 1B) indicates that

(1) $$k_{\text{SW}}\text{d}x+\Phi +\text{d}\Phi =k_{0}\text{d}x\text{ sin }\theta_{r}+\Phi \text{,}$$

where Φ and Φ+dΦ are the abrupt phase shifts encoded by meta-atoms at two adjacent positions *x* and *x* + d*x*, respectively. Equation (1) can be re-derived as $k_{0}\text{ sin }\theta_{r}=k_{\text{SW}}+\xi$ with $\xi =\frac{\text{d}\Phi }{\text{d}x}$ being the phase gradient of the meatsurface, implying that an impinging SW can transformed to a free-space PW with a tangential wave-vector $k_{x}=k_{\text{SW}}+\xi$ smaller than $k_{0}$ (where ξ should be a negative value). The orientation angles of our meta-atoms can be easily derived as α(x)=ξ⋅x/2 . Interestingly, we note that the abnormally scattered waves exhibiting right circular polarization (RCP) can see an opposite phase gradient and their interference can only form an evanescent wave with a tangential wave-vector $k_{x}=k_{\text{SW}}-\xi$ (noting that ξ<0 ), which cannot form a far-field PW and will be eventually converted to the eigen SWs supported by the device. Compared with Bragg devices, such meta-devices can modulate the target wavefronts in deep-subwavelength scales thus avoiding multi-mode generations and exhibiting high working efficiency, and can generate spin-polarized far-field beams based on designing the α(x) profile.

**Figure 2:**
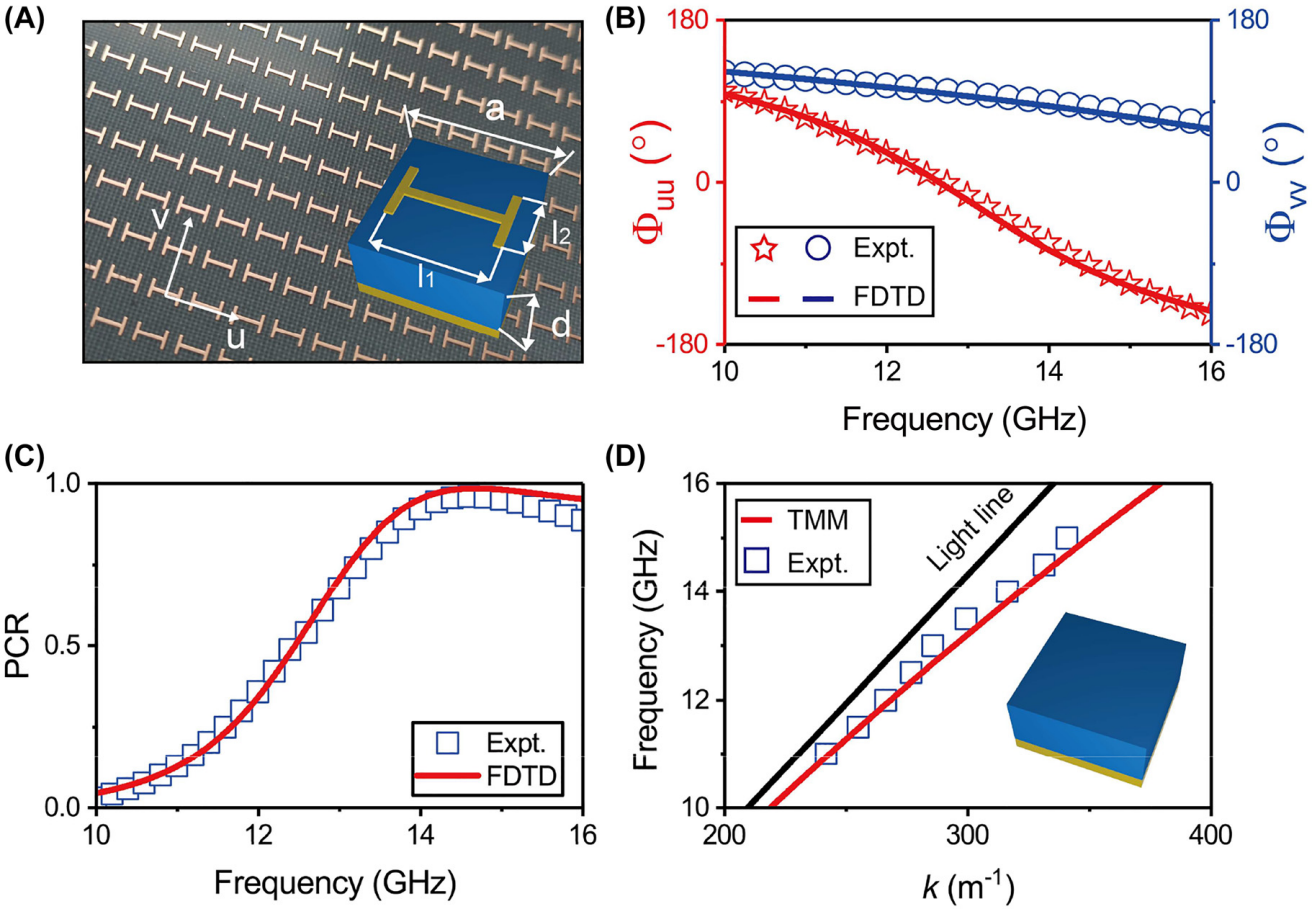
Characterizations of the designed PB meta-atoms and plasmonic system. (A) Picture of the fabricated PB meta-atoms composed by the copper H-shaped resonator and a copper film that are separated by a dielectric spacer $\left(\epsilon \prime =3\right)$ . Here, *a* = 4.8 mm, *d* = 2 mm, *l*_1_ = 3.5 mm and *l*_2_ = 2 mm. (B) and (C) Reflection phase ( $\Phi_{uu}$ and $\Phi_{vv}$ ) and PCR spectra of designed meta-atoms obtained by simulations and experiments. (D) Dispersion relation of the eigen SWs supported by the plasmonic metal depicted in the inset, based on numerical calculations and near-field measurements.

As a proof of concept, we choose the microwave regime to demonstrate our idea. Figure 2A depicts the proposed/fabricated PB meta-atom in metal–insulator–metal (MIM) configuration, consisting of an H-shaped copper resonator and a copper thin film separated by a 2 mm-thick F4B dielectric spacer $\left(\epsilon_{r}=3\right)$ . We first study the reflection property of such anisotropic unit structure under normal incidence, which can be described by a Jones matrix $R=\left(\begin{array}{ll} r_{uu} & 0\\ 0 & r_{vv} \end{array}\right)$ with u and v denoting two principle axes of the meta-atom. Figure 2B depicts the spectra of reflection phases $\Phi_{uu}$ and $\Phi_{vv}$ of a periodic array of such meta-atoms, obtained by both numerical simulations and experimental measurements. We note that the phase-difference $\Phi_{uu}-\Phi_{vv}$ increases continuously from $28^{\circ }$ to $191^{\circ }$ in the considered frequency window. On the other hand, the reflection amplitudes $\left|r_{uu}\right|$ and $\left|r_{vv}\right|$ are both 100% since the bottom copper mirror behaves as a perfect metal which can totally reflect the impinging EM waves. Defining ${\left|\left(r_{uu}-r_{vv}\right)/2\right|}^{2}$ as the PCR of the meta-atom, we find that PCR reaches 100% at 14.5 GHz (see Figure 2C), at which the PB meta-atom behaves as an ideal half wave plate that can completely flip the spin state of impinging circular polarization (CP) waves [52], [53], [54]. According to the theory presented in Ref. [52], we find that the amplitude of the abnormally scattered waves is proportional to the square root of PCR. Therefore, via changing the meta-atom structure or varying the working frequency, we can control the amplitude of abnormally scattered wave, which is an additional important degree of freedom to control the generated far-field wavefront based on our scheme (see Section C of Supplementary Material).

## 3 Unidirectional far-field radiation

**Figure 3:**
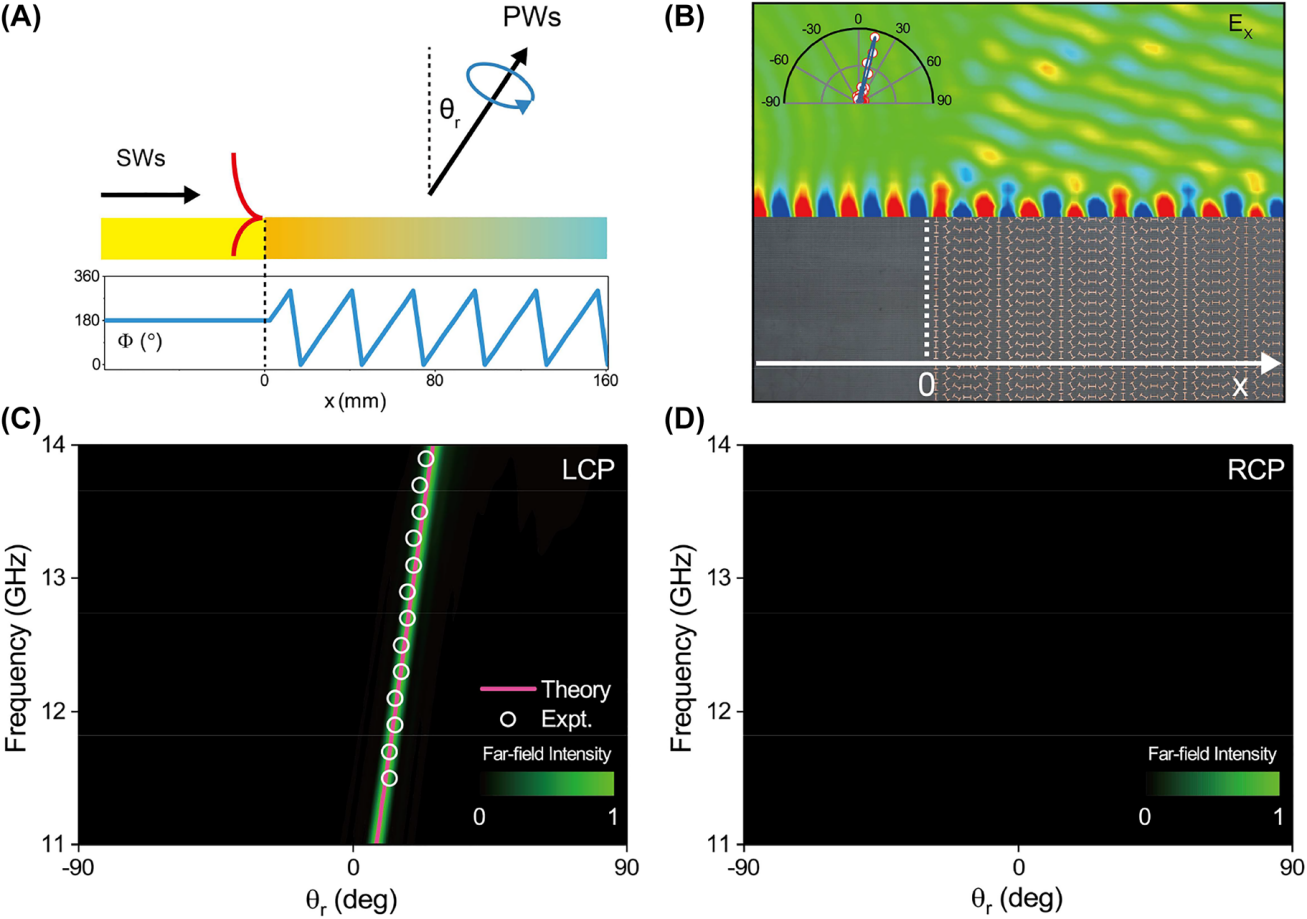
Characterizations of SW–PW unidirectional radiation based on PB metasurface. (A) Schematic of the plasmonic metal (left) and PB metasurface with a linear phase profile (right) for SW–PW radiation. (B) Measured *E*_*x*_ field distribution of the sample (see bottom figure) for SW–PW radiation at 12 GHz, obtained by the near-field mapping technique. The inset figure illustrates the simulated (blue line) and measured (red open circles) scattered far-field angular distribution at 12 GHz. (C) and (D) The scattered far-field intensities of (C) LCP and (D) RCP radiated by our device as functions of radiation angle and frequency.

We now investigate the unidirectional far-field emission from SW excitations on the gradient PB metasurface, as illustrated in Figure 3A. We first design a “plasmonic metal” supporting eigen SWs, so that an impinging SW can be excited as the source. Without causing too much undesired scatterings, the “plasmonic metal” that we design is simply a metallic ground plane covered by a dielectric spacer of the same thickness as that of the PB meta-atom (see inset to Figure 2D). Dispersion relation of SWs supported by such a structure can be computed analytically, which is further verified by our near-field experimental measurements on a realistic sample (see Figure 2D). Choosing the working frequency as 12 GHz, we find that our “plasmonic metal” supports an eigen SW with $k_{\text{SW}}=1.07k_{0}$ . We then design a gradient PB metasurface composed by a series of identical PB meta-atoms with orientation angles varying in a step Δα=30° , providing a phase gradient of $\xi_{1}=-0.87k_{0}$ . The final device consists of the designed PB metasurface connected with a “plasmonic metal”, as shown in Figure 3B. According to Eq. (1), as we launch an eigen SW on the left-sided plasmonic metal to flow across the metasurface, we expect that the scatterings by the metasurface will form a PW radiating along the angle $\theta_{r}=14{}^{\circ}$ . To verify our prediction, we design and fabricate the device (see Figure 3B), and then perform near-field mapping technique to measure the field distribution on an *x*–*z* plane. Our experimental results demonstrate that, as the source SW (excited by a meta-coupler under the illumination of a normally incident PW, see more details in Section D of Supplementary Material) travels through the PB metasurface, scatterings by meta-atoms form a free-space PW propagating along the pre-designed direction. We note that the meta-atom exhibits a PCR ∼ 0.4 at the working frequency 12 GHz (see Figure 2C), indicating that the decoupling of SW to PW is relatively weak (or the intensity decay of SW is slow) as it flows on the metasurface. As a result, different meta-atoms inside the meta-device are excited by SWs exhibiting approximately identical strength, so that the generated far-field wavefront is quite flat (see Figure 3B). It is also noted that both diffraction (about 1.7%) and reflection (less than 1%) are negligible at the interface between the plasmonic metal and the PB metasurface, since their impedance (for SWs) mismatch is very small [50, 55], [56], [57]. Rotating a CP receiver antenna on a 1 m-radius circle with the sample as center, we further measure the angular distributions of scattered far-field intensity at different frequencies, normalized against the maximum values of scattered intensities at the corresponding frequencies. Both experimental and simulation results perfectly verify the theoretical prediction of single-mode directional scattering along $\theta_{r}=14{}^{\circ}$ at 12 GHz (see Figure 3B).

We experimentally characterize the polarization state of the generated beam. Adopting the LCP antenna as the receiver, we find that the measured normalized angular distributions of scattered field agree very well with theoretical prediction given by Eq. (1) (see pink solid line in Figure 3C), exhibiting nice single-mode emission within the frequency band (11–15 GHz). As we change to use the RCP receiver antenna, we cannot collect any far-field emissions (see Figure 3D). These findings undoubtedly demonstrate that the out-going PW generated by our PB device carries a pure LCP. Moreover, the polarization characteristic the radiated PW beam can be well controlled by changing the orientation sequence of PB meta-atoms in the device (see Section E in Supplementary Material).

**Figure 4:**
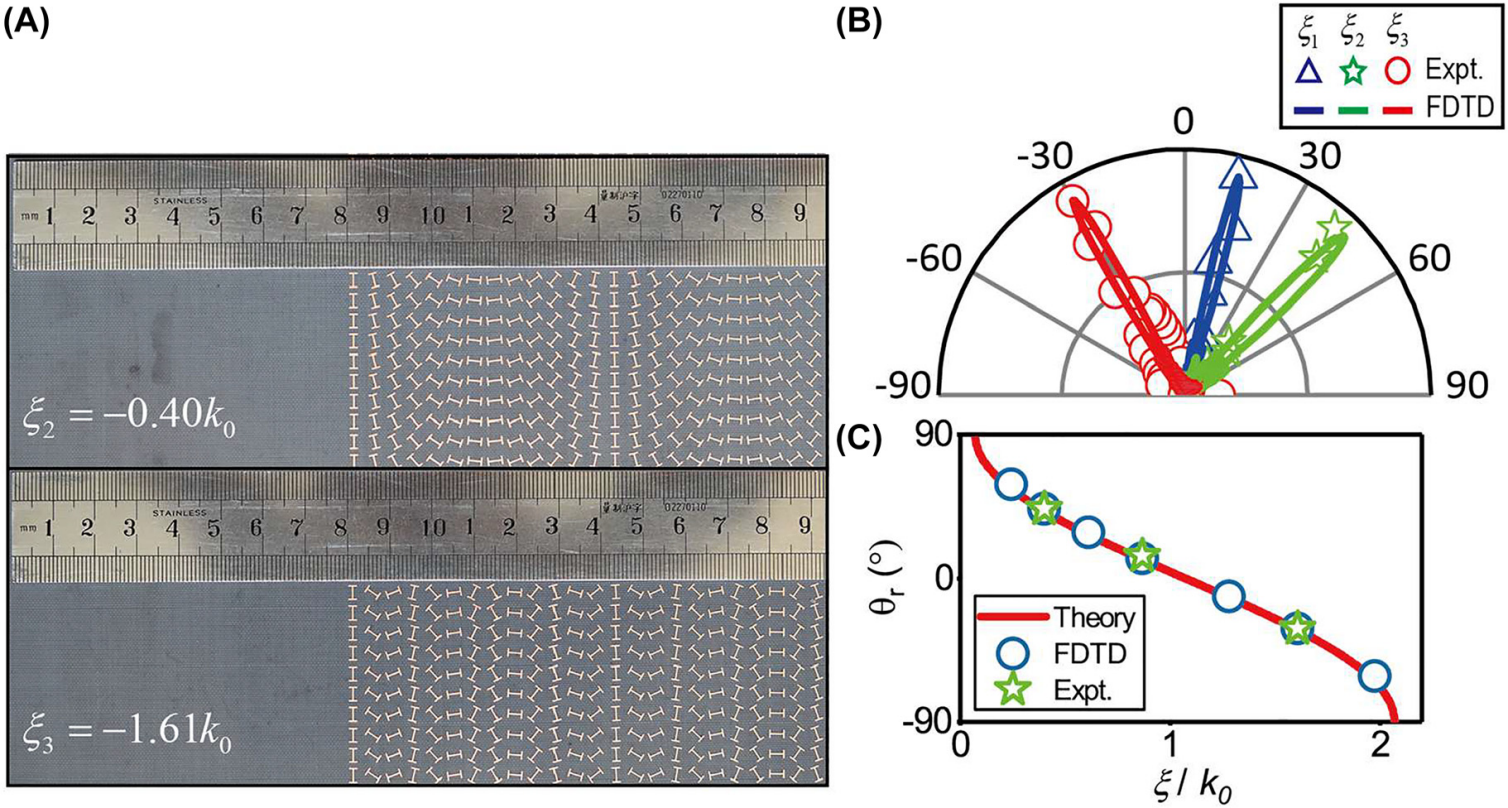
Verification of SW–PW unidirectional radiation covering full radiation angle. (A) Sample images of two meta-devices with different phase gradients: $\xi_{2}=-0.40k_{0}$ and $\xi_{3}=-1.61k_{0}$ . (B) The measured (solid lines) and simulated (open dots) scattered far-field angular distributions for three different meta-devices of different phase gradient. (C) Radiation angle as a function of phase gradient of the adopted metasurface for SW–PW conversion. Here, the frequency is fixed at 12 GHz.

We can freely modulate the radiation direction covering the full angle range ( −90° to +90° ) via changing the phase gradient of our PB metasurface. Figure 4A depicts two more fabricated samples with phase gradients $\xi_{2}=-0.40k_{0}$ and $\xi_{3}=-1.61k_{0}$ , respectively. Redoing the same experiments and simulations as in the case of sample 1, we find that the three devices can generate out-going PWs traveling to $\theta_{r}=-30{}^{\circ},14{}^{\circ},45{}^{\circ}$ (see Figure 4B), as excited by an impinging SW at 12 GHz. Meanwhile, numerically simulated electric-field distributions of such processes also verify the single-mode conversions based on our fabricated PB metasurfaces (see Section F in Supplementary Material). In fact, we have designed a series of PB metasurfaces exhibiting different phase gradients and numerically characterized their capabilities to decouple impinging SWs to PWs. The propagating directions of the generated PWs fully agree with theoretical predictions (see Figure 4C).

**Figure 5:**
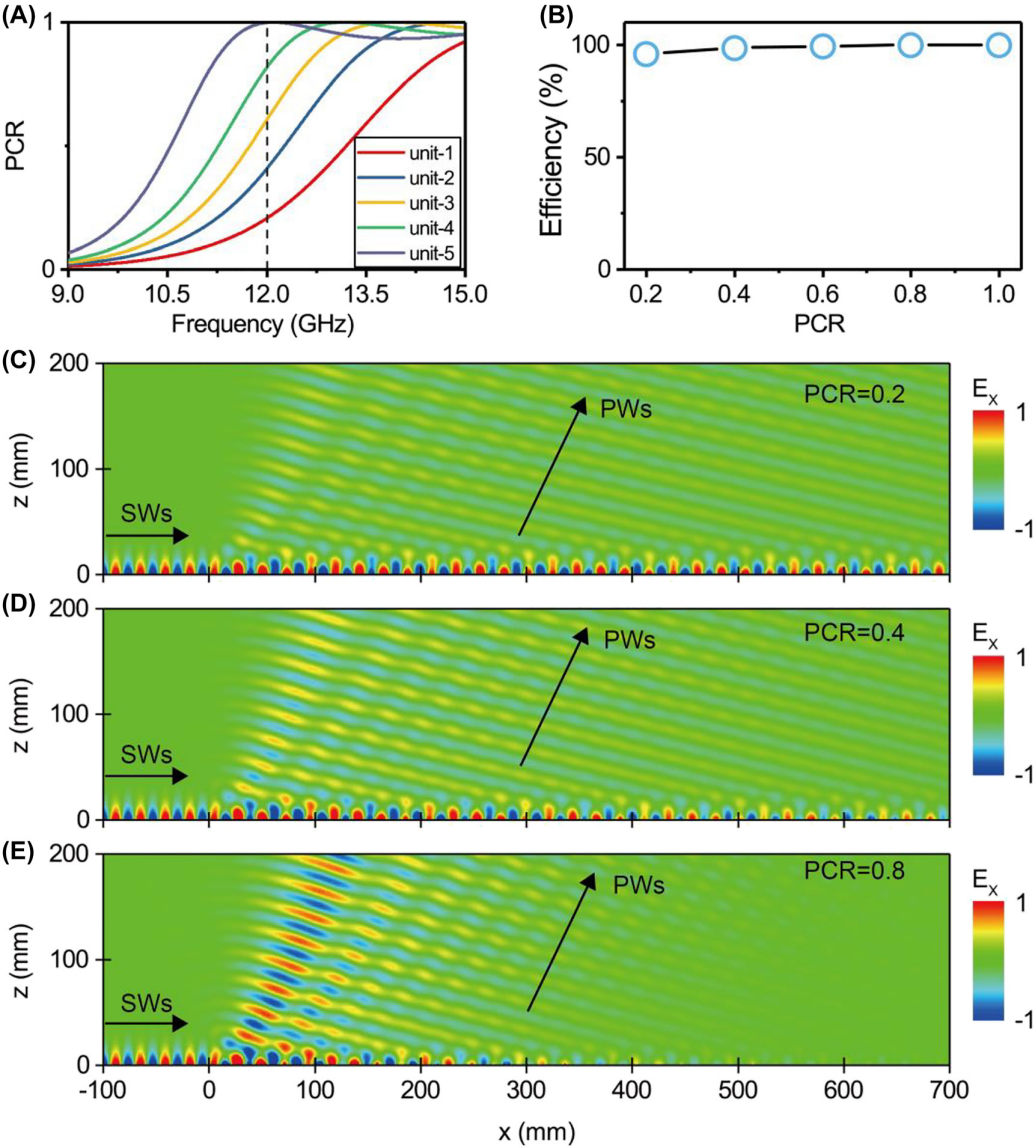
Decoupling speed and conversion efficiency of SW–PW radiation based on PB metasurfaces of different PCRs. (A) PCR as a function of frequency for five different PB unit structures with different *l*_2_ (*l*_2_ = 1.6, 2.0, 2.25, 2.5, 2.9 mm). All of the other parameters are totally same to those of the meta-atoms in Figure 2A. (B) Conversion efficiency of SW–PW radiation as a function of PCR for the adopted PB metasurface, computed based on full wave simulations. (C)–(E) Simulated *E*_*x*_ field distribution of SW–PW radiation with PB metasurface of the same phase gradient but different PCRs (i.e., 0.2, 0.4, 0.8). Here, the metadevice consists of 150 meta-atoms (i.e., 720 mm), which is long enough to decouple all impinging SWs.

We discuss how the PCR of the constituent meta-atom influences the performance of PB meta-device. We design five different PB meta-atoms exhibiting distinct PCR values at the frequency 12 GHz, via simply tuning the parameter $l_{2}$ from 1.6 to 2.9 mm (see Figure 5A). Employing these meta-atoms to construct five PB meta-devices with the same phase gradient $\xi =-0.87k_{0}$ , we numerically studied their performances under the same SW excitations at 12 GHz. Figure 5C–E compares the simulated *E*_*x*_ field distributions to illustrate the unidirectional emissions in three systems with PCR = 0.2, 0.4, 0.8, respectively. In all different cases, the generated PWs are travelling along almost the same direction considering that the change of $l_{2}$ has neglectable influence on the wavevector of SW (as depicted in Figure 5C–E). However, we find that the flatness and the width of generated PW beam sensitively depends on the PCR value of the constituent meta-atom. In the case of a larger PCR value, the generated PWs are of a smaller beam width since the decoupling rate of SW–PW increases. Meanwhile, waves scattered by the PB meta-atoms located at positions deep inside the device may exhibit much weaker strength than that generated by the firstly excited meta-atom, implying that the flatness of radiation beam wavefront become worse. Nevertheless, we find that final SW–PW conversion efficiency remains stable as long as the meta-device (consisting of 150 meta-atoms, i.e., 720 mm long) exhibits a large enough length to decouple all impinging SWs. We have calculated the SW-PW conversion efficiencies (defined as the ratio between integrated power of the radiated PW and that of the impinging SW) of our PB meta-devices, and show the calculated efficiency verse PCR in Figure 5B. The SW–PW conversion is found to be close to 100%, nearly independent of the PCR value. We note that this is a unique feature of presently discussed SW–PW process, being quite different from its time-reversal counterpart (i.e., the PW–SW conversion) studied in previously literature, where the conversion efficiency will be proportional to the PCR of constituent meta-atoms [26, 28].

We discuss an important advantage of our metasurface-based scheme to achieve pre-designed far-field scattering patterns. As shown in previous discussions, meta-atoms inside our PB meta-devices can control not only the phases of locally scattered waves (dictated by the orientation angles of meta-atoms), but also the amplitudes of such waves (dictated by the PCR values of meta-atoms), all in deep-subwavelength scale. It has been shown (see Figure 5E) that the generated far-field beam does not exhibit a flat wavefront in the large-PCR meta-atom case. This can be remedied by slightly adjusting the meta-atoms structures at different locations (see numerical demonstration in Figure S7 of Supplementary Material), so that their SW–PW scattering capabilities (related to their PCR values) can compensate the decrement in local SW strength due to scattering losses of SW flowing on the device surface. Such local-control capabilities on both phase and amplitude offer us possibilities to manipulate the far-field radiation patterns as desired with deep-subwavelength resolution, which can find numerous photonic applications [58].

## 4 Arbitrary scattering far-field patterns

**Figure 6:**
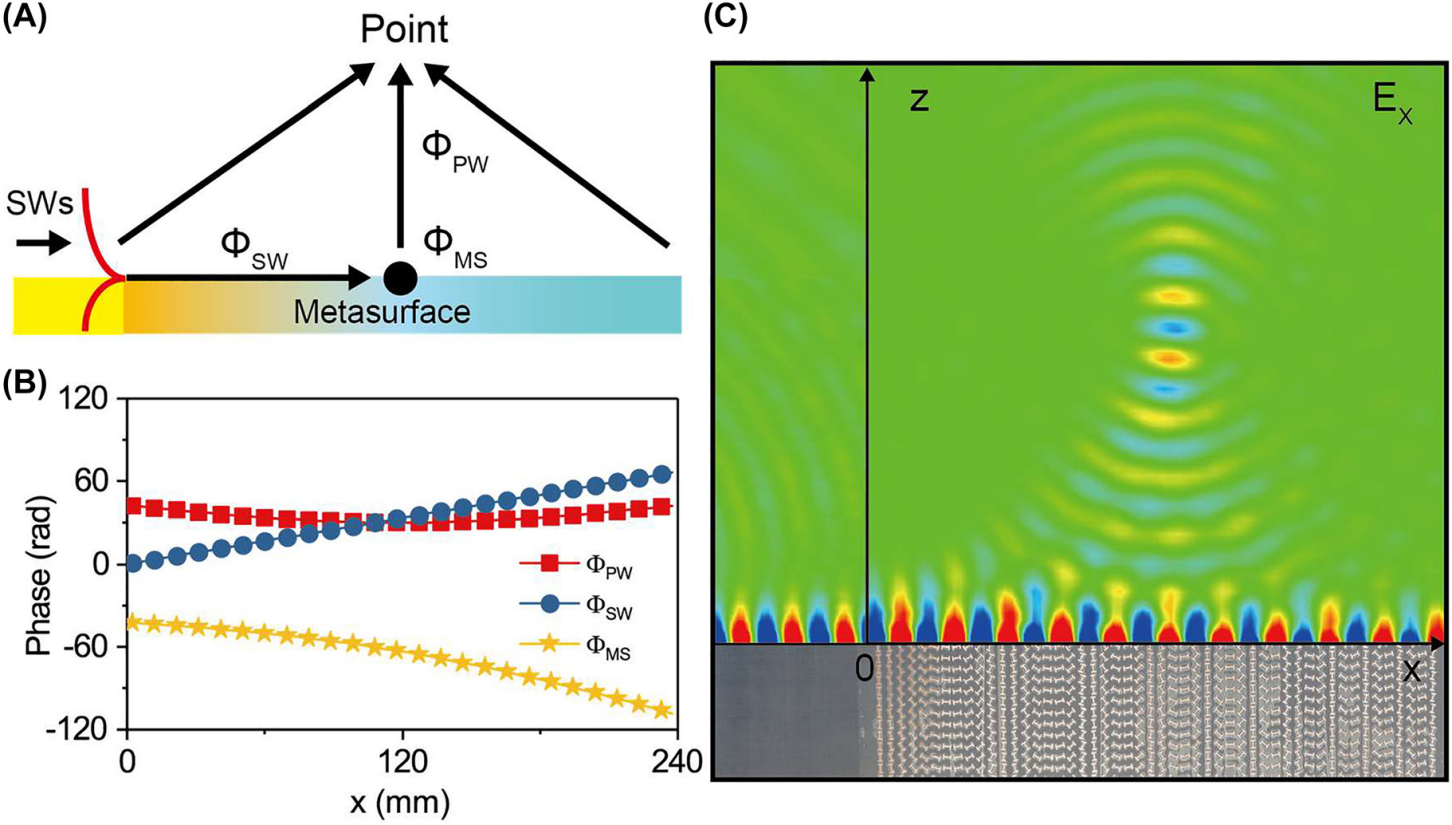
Far-field line focusing of SWs decoupled by the metasurface. (A) Schematic of line focusing effect of SW–PW radiation with metasurface. (B) Three parts of phases, i.e., the propagation phase of SWs on metasurface $\Phi_{\text{SW}}$ , the abrupt phase shift $\Phi_{\text{MS}}$ and the propagation phase of PWs in air $\Phi_{\text{PW}}$ , through different optical path of the PW emitted from the substructures at different local positions. (C) The measured *E*_*x*_ field distribution in our meta-device for achieving line focusing of SW–PW radiation.

We use the proposed strategy to demonstrate other meta-devices to achieve more complicated scattering far-field patterns. The first example is line focusing as depicted in Figure 6A. Based on the similar analyses as in Section 2, we find that the phase profile of the PB meta-device Φ(x) should satisfy

(2) $$k_{\text{SW}}x+k_{0}r+\Phi \left(x\right)=0\text{,}$$

where $k_{\text{SW}}x$ denotes the propagation phase of excitation SW, $k_{0}r$ denotes the propagation phase of radiated PW in free space (with $r=\sqrt{F^{2}+{\left(x-L/2\right)}^{2}}$ ) with *L* being the total length of the device and *F* the designed focal length. Again, we assume that our meta-device is working for LCP polarization, and thus we can easily retrieve the orientation-angle profile α(x) of meta-atoms inside the device. Setting *L* = 240 mm, *F* = 120 mm and the central working frequency as 12 GHz, we adopt the same meta-atom studied in Section 2 as our building block and construct the meta-device based on the retrieved α(x) function. Figure 6C depicts the picture of the fabricated sample. We then perform near-field mapping experiment to measure the $E_{x}$ distribution on an *x*–*z* plane inside the system, under the excitation of an SW launched at 12 GHz. Figure 6C clearly shows that the incident SW has indeed been scattered to a focal line at about 125 mm above the device. Full-wave simulation results (see Figure S8A in Supplementary Material) are in good agreement with experimental results. To improve the desired focusing effect, we again adjusted the PCRs of meta-atoms at different positions in designing our PB metasurface (see Section G in Supplemental Material for more discussions).

**Figure 7:**
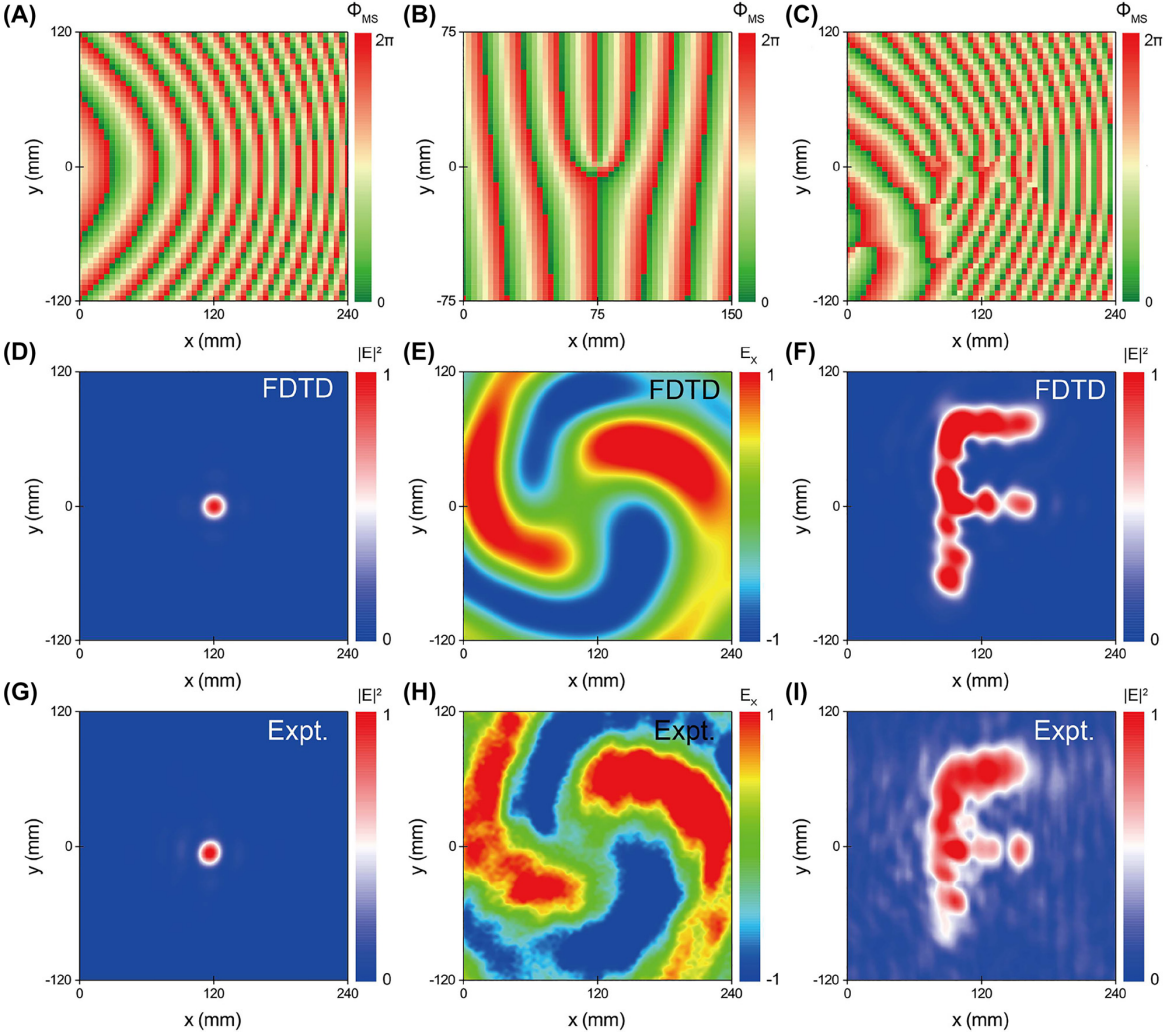
Complicated scattering far-field patterns with PB metasurfaces. (A), (D), (G) Far-field 3D focusing, (B), (E), (H) vortex beam and (C), (F), (I) hologram are generated by different SW metasurfaces according to Eq. (3). Scattered far-field patterns (on *x*–*y* plane) excited by SWs with three different PB metasurfaces, obtained by (D)–(F) experimental measurements and (G)–(I) full wave simulations. (A)–(C) The phase profiles of PB metasurfaces to achieve three functionalities. Here, the frequency is fixed at 12 GHz.

We use the proposed strategy to further demonstrate, both numerically and experimentally, three different meta-devices, that can generate far-field scattering patterns including three-dimensional (3D) focusing, vortex beam and hologram (see Figure 7), respectively. Based on the same analyses, we find that the phase profiles of these devices should satisfy

$$\left\{ \begin{array}{ll} k_{\mathrm{SW}}x+\Phi \left(x,y\right)=-k_{0}\sqrt{F^{2}+{\left(x-L/2\right)}^{2}+y^{2}} & \;\left(3a\right)\\ k_{\mathrm{SW}}x+\Phi \left(x,y\right)=q\text{ arctan}\left[y/\left(x-L/2\right)\right] & \;\left(3b\right)\\ k_{\mathrm{SW}}x+\Phi \left(x,y\right)=\Phi_{\mathrm{CGH}} & \;\left(3c\right) \end{array}\right.$$

respectively.

We design and fabricate the first meta-device for achieving 3D focusing (with *F* = 120 mm and *L* = 240 mm) with 50×50 identical PB meta-atoms and the same “plasmonic metal” as studied in Section 2, according to the phase distribution Eq. (3a) (see Figure 7A). FDTD simulations and near-field measurements perfectly verify the predicted focusing effect with focal point on the plane *z* = 120 mm (see Figure 7D and G), as the device is excited by an SW at 12 GHz.

We next design the second meta-device based on the phase profile shown in Eq. (3b), which is expected to generate a vortex beam exhibiting orbital angular momentum (OAM) with a topological charge *q* = 2. Here, different from the first device, we have individually designed meta-atoms at different locations to make them exhibit particular PCR values, so that locally scattered waves can roughly exhibit the same amplitude. In addition, lattice spacing between adjacent PB meta-atoms is also reduced to improve the subwavelength properties. Figure 7B depicts the desired phase distribution possessed by the meta-device. We then fabricate the device and experimentally characterize its functionality at 12 GHz. Measured *E*_*x*_ field distribution on the plane of *z* = 400 mm clearly demonstrates the OAM beam generation with *q* = 2, which also agrees well with FDTD simulation results (see Figure 7E and H).

Our scheme is so generic that we can also use it to realize holographic images in free space, with meta-devices exhibiting carefully designed phases (see Eq. (3c)). As a proof of concept, we design the third meta-device to realize a free-space image of a letter ‘F’ on the plane of *z* = 120 mm, as the device is excited by an SW at 12 GHz. To achieve this goal, we first adopt the Gerchberg–Saxton (GS) algorithm [59], [60], [61] to retrieve the PB phase distribution $\Phi_{\text{CGH}}$ needed by a metasurface to realize the desired image as it is shined by normally incident PW. With $\Phi_{\text{CGH}}$ known, we then add the SW propagation-phase term $k_{\text{SW}}x$ (see Eq. (3c)) to obtain the desired phase distribution Φ(x,y) of the meta-device under SW excitation. We finally design and fabricate the meta-device according to the phase distribution Φ(x,y) , and use the same techniques to characterize the functionality of the device under SW excitation, both numerically and experimentally. Both simulated and experimentally measured field pattern on the plane of *z* = 120 mm clearly show an image of “F” (Figure 7F and I), well verifying the theoretical prediction.

## 5 Conclusions

In this work, we propose a generic strategy to design PB meta-devices for achieving spin-polarized far-field scattering wavefronts, and experimentally verify the concept in the microwave regime. Under SW excitations, carefully designed PB meta-atoms can scatter impinging SWs to spin-polarized free-space waves with tailored amplitudes and phases, and thus their interference can form arbitrary scattering far-field patterns in subwavelength scales. A series of meta-devices are designed, fabricated and characterized, which can realize far-field patterns including unidirectional emission, 2D and 3D focusing, OAM beam and hologram. Compared to other meta-devices for controlling PWs based on PW excitations [24, 62], present meta-devices show the advantages of high-integration and high-efficiency, which are highly desired for future on-chip photonic applications. However, different local fields experienced by different meta-atoms inside device also obviously increase the complexity in designing these meta-devices. After realizing high-efficiency PW-SW coupling in our previous works [24, 63], we have finally achieved the two-way conversions between PW and SW, significantly expanding our capabilities to manipulate EM waves. Such a concept is quite generic that can be further developed to high frequency regimes. Our findings may inspire important applications in integration optics, such as leaky antenna, virtual reality imaging, plasmonic micro projector, virtual reality displays, etc., and realizing them in different frequency regimes are interesting future projects.

## Supplementary Material

Supplementary Material
