# Supplementary material for: High-efficiency generation of far-field spin-polarized wavefronts via designer surface wave metasurfaces
Source: Nanophotonics. 2022 Mar 15;11(9):2025–36. doi: 10.1515/nanoph-2022-0006 (PMC11501909; doi:10.1515/nanoph-2022-0006)
Supplement: Supplementary file 1 — Supplementary Material [file j_nanoph-2022-0006_suppl.docx]

**Supplementary Material**

**High-efficiency generation of far-field spin-polarized wavefronts via designer surface wave metasurfaces**

Weikang Pan^1,3, a^, Zhuo Wang^2,a^, Yizhen Chen^1^, Shiqing Li^1^, Xiaoying Zheng^2^, Xinzhang Tian^1^, Cong Chen^4^, Nianxi Xu^5^, Qiong He^2,6^, Lei Zhou^2,6,*^, Shulin Sun^1,3,*^

^1^*Shanghai Engineering Research Center of Ultra-Precision Optical Manufacturing,* *Department of Optical Science and Engineering, School of Information Science and Technology, Fudan University, Shanghai 200433, China*

^2^*State Key Laboratory of Surface Physics and Key Laboratory of Micro and Nano Photonic Structures (Ministry of Education), Fudan University, Shanghai 200433, China*

^3^*Yiwu Research Institute of Fudan University, Chengbei Road, Yiwu City, 322000 Zhejiang, China*

*^4^School of Electronic Information, Wuhan University, Wuhan 430072, China*

^5^*Changchun Institute of Optics, Fine Mechanics and Physics, Chinese Academy of Sciences, Changchun, Jilin 130033, China*

^6^*Collaborative Innovation Center of Advanced Microstructures, Nanjing 210093, China*

Keywords: surface wave; propagation wave; far-field wavefront; Pancharatnam-Berry phase; metasurface

^a^ Weikang Pan and Zhuo Wang are co-first authors.

*Corresponding authors: Shulin Sun, [sls@fudan.edu.cn](mailto:sls@fudan.edu.cn); Lei Zhou: [phzhou@fudan.edu.cn](mailto:phzhou@fudan.edu.cn).

**Outline**

[A. Multi-mode generations in SW-PW conversion with Bragg structures 3](#_Toc92323789)

[B. Radiations of a single meta-atom and PB metasurface excited by SWs 4](#_Toc92323790)

[C. Radiation amplitude of SW modulated by the PCR of meta-atom 5](#_Toc92323791)

[D. Details of the SW meta-coupler adopted in our experiments 6](#_Toc92323792)

[E. Different spin-polarized far-field emissions of SWs 7](#_Toc92323793)

[F. Far-field emissions of SWs with PB metasurfaces of different phase gradients 8](#_Toc92323794)

[G. Far-field radiations of SWs with PB metasurfaces of inhomogeneous PCRs 9](#_Toc92323795)

# A. Multi-mode generations in SW-PW conversion with Bragg structures

**
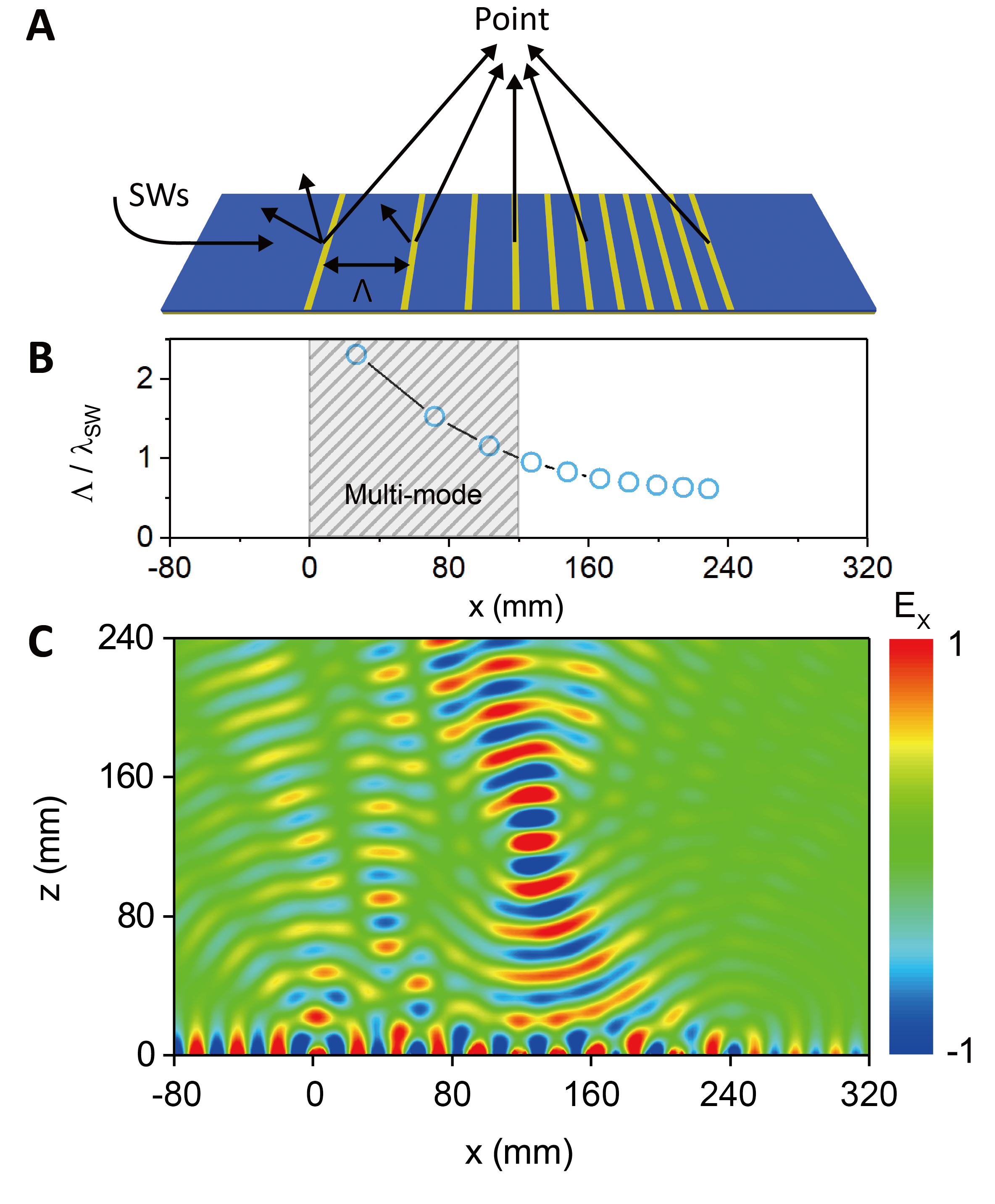
**

FIG. S1: (A) Schematic of far-field focusing based on SW-PW radiation with inhomogeneous Bragg grating. (B) The local periodicity $\Lambda$ at different positions of inhomogeneous Bragg grating to achieve SW-PW focusing. Multiple scattering modes will appear in the shadow area of inhomogeneous grating satisfying$\Lambda/\lambda_{SW}\geq2\lambda_{0}/(\lambda_{0}+\lambda_{SW})$. (C) Simulated E_x_ field distribution of SW-PW radiation in the system depicted in A, exhibiting the multipole mode generation effect. Here, the total size (240 mm) and focal length (F=120 mm) of the inhomogeneous grating are totally same to those of the corresponding PB metasurface shown in Figure 6. And the frequency is 12 GHz.

# B. Radiations of a single meta-atom and PB metasurface excited by SWs

**
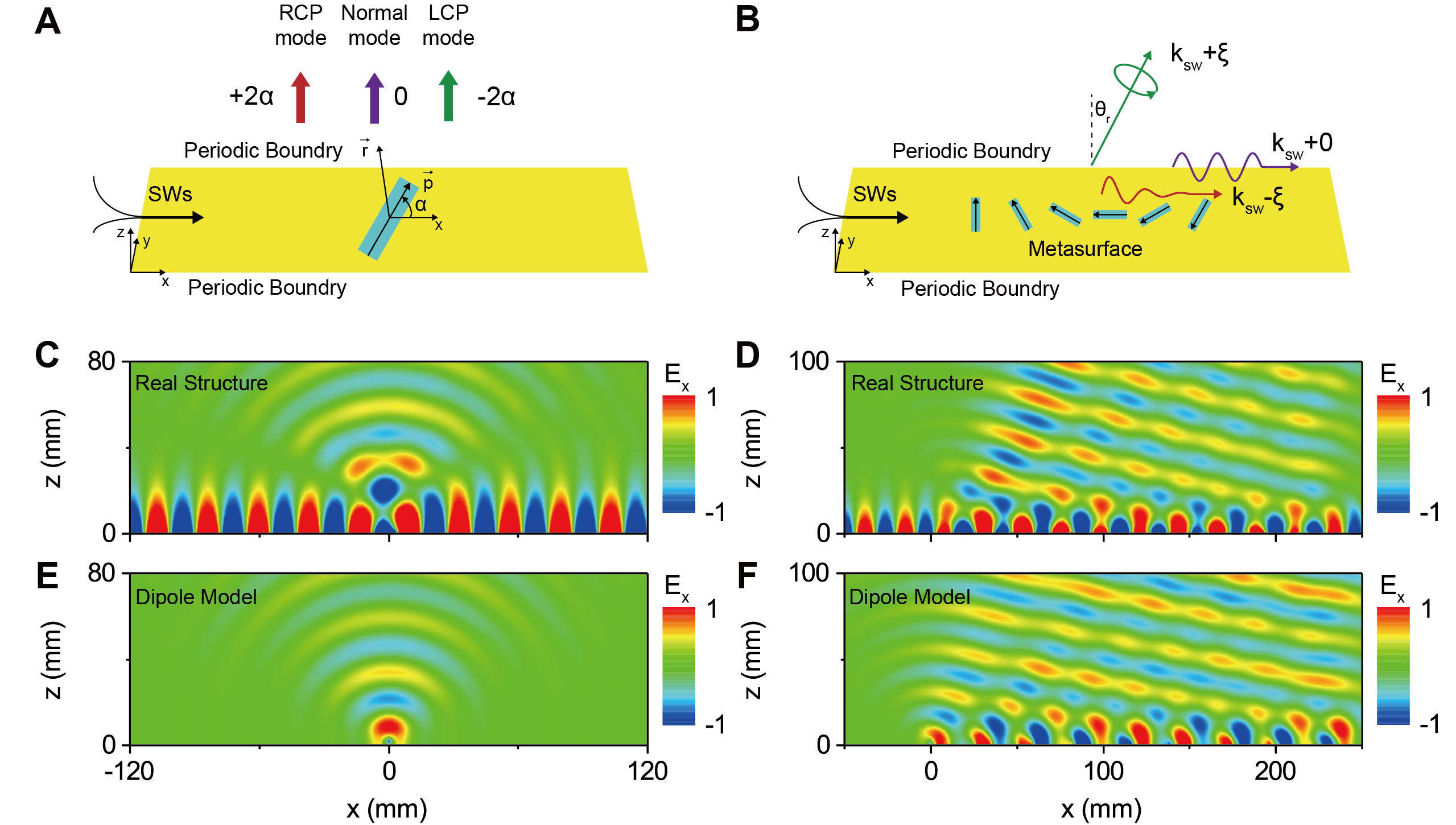
**

FIG. S2: (A) Schematic of far-field radiation of a single meta-atom under the excitation of SWs, which will generate one normal mode (purple arrow) without geometric phase response and two anomalous modes (red and green arrows) carrying spin dependent geometric phase $\pm2\alpha$. (B) Schematic of far-field radiations of a PB gradient metasurface under the excitation of SWs. Interference of the anomalous LCP modes generated by different PB meta-atoms will lead to SW-PW directional radiation according to the equation $k_{0}\sin\theta_{r}=k_{SW}+\xi$ (noting that $\xi<0$). The other two kind of modes cannot provide the desired phase gradient to decouple the impinging SW to free space PW. (C, D) The simulated $E_{x}$ field distributions of a single meta-atom and a PB metasurface (consisting of 50 meta-atoms with their orientation angles varying in a step $\Delta\alpha=30^{\circ}$). (E, F) The calculated $E_{x}$ field distributions of a single dipole and 50 discrete dipoles with spatially varied orientations ($\Delta\alpha=30^{\circ}$) and phases ($\Delta\Phi=k_{SW}a\text{=74}^{\circ}$). Here, the lattice constant (*a*) of realistic meta-atoms and discrete dipole array is 4.8mm. The frequency is 12 GHz.

# C. Radiation amplitude of SW modulated by the PCR of meta-atom


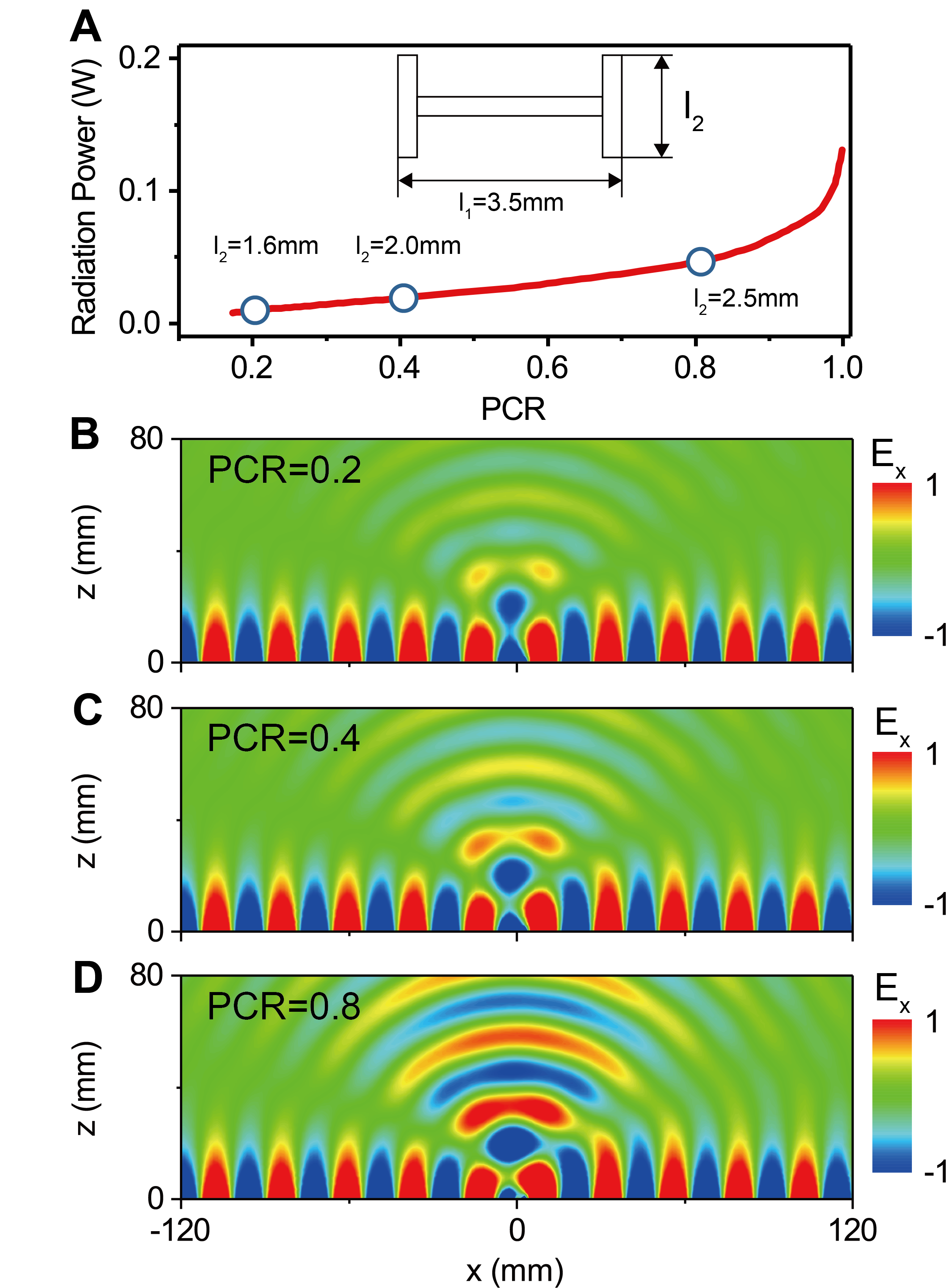


FIG. S3: (A) Integrated radiation field power of a single PB meta-atom excited by the impinging SWs as a function of its PCR (via tuning *l_2_*). (B-D) Simulated scattered field distributions in the x-z plane for three special cases, i.e., PCR= 0.2, 0.4, 0.8. Here, the input power of SWs is 1W and the frequency is 12 GHz.

# D. Details of the SW meta-coupler adopted in our experiments


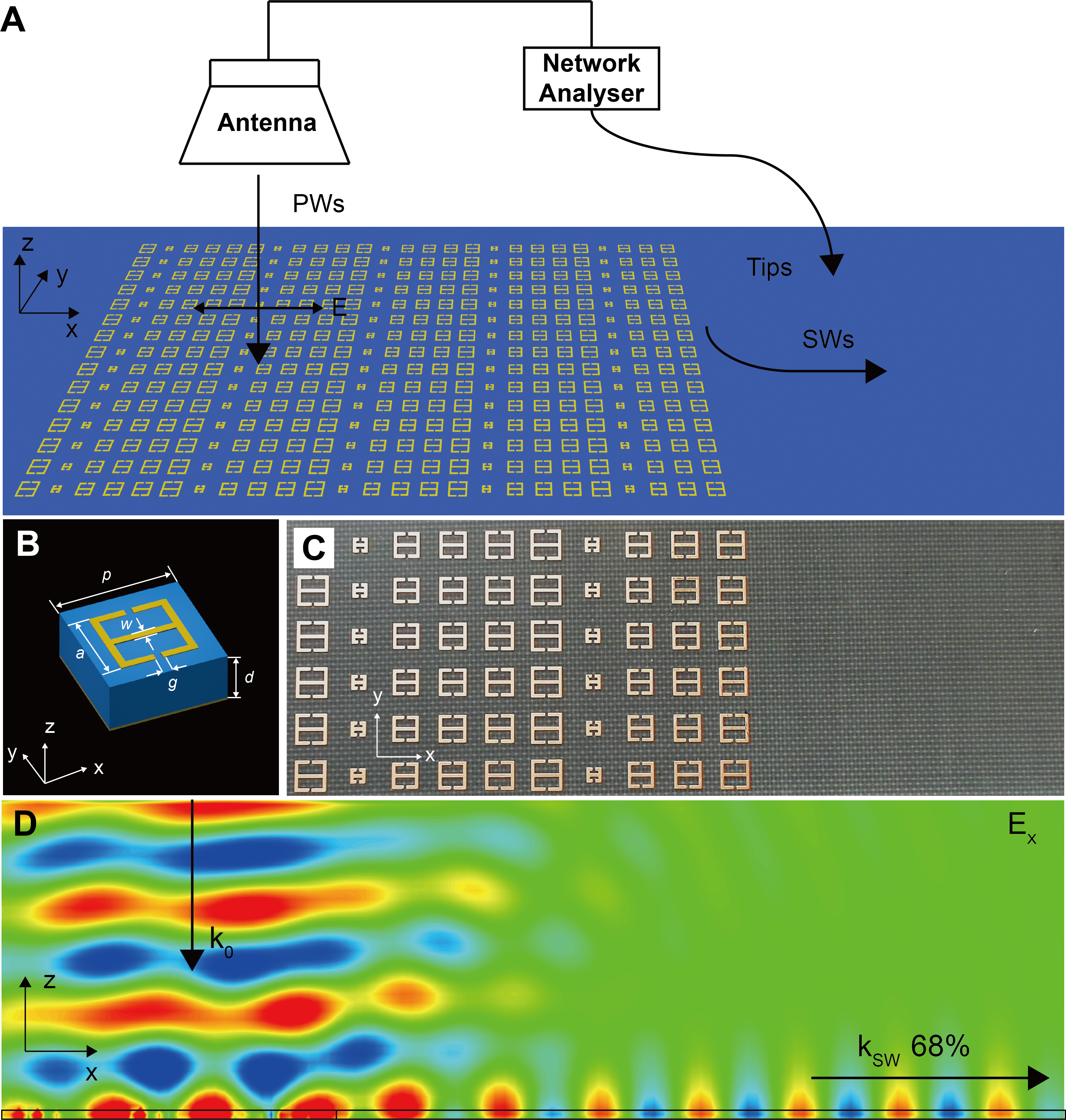
FIG. S4: Schematic (A) and sample picture (C) of the adopted SW meta-coupler in our microwave experiments. (B) Schematic of a unit cell consisting of a H-shaped metallic microstructure and a PEC mirror separated by a dielectric spacer ($\varepsilon=3$). One super cell of the meta-coupler is composed by 5 sub cells with the arm length *a* varied as 1.5mm, 2.63mm, 2.80mm, 2.92mm, 3.15mm, respectively. The other parameters are fixed: *p*=4.8mm, *w*=0.3mm, *g*=0.3mm, *d*=2mm. (D) Simulated *E_x_* distributions inside the meta-coupler illuminated by the normally incident PWs at 12 GHz. The conversion efficiency of PW-SW is about 68%.

# E. Different spin-polarized far-field emissions of SWs

**
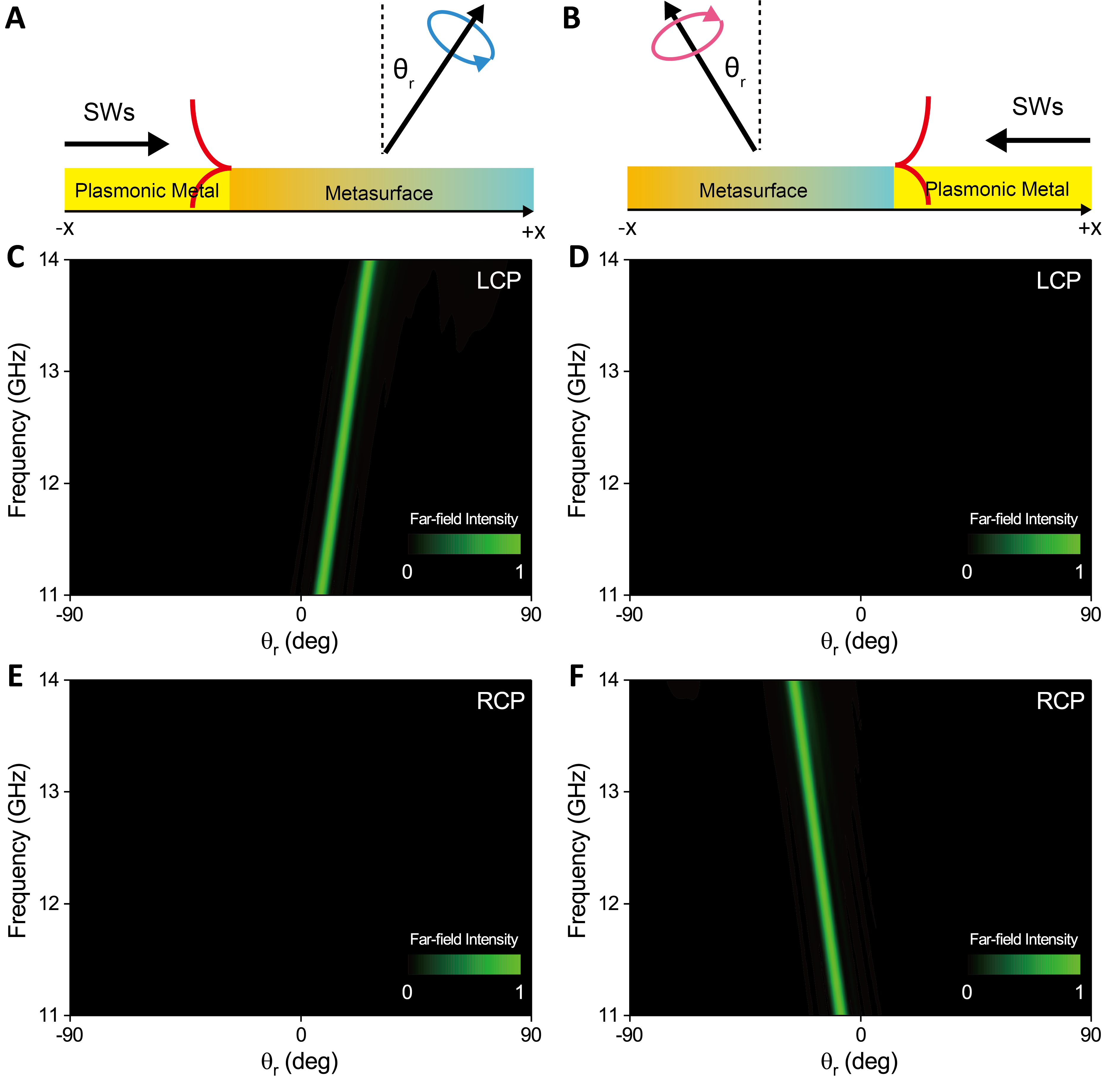
**

FIG. S5: Normalized scattered far-field intensities of (C, D) LCP and (E, F) RCP as functions of radiation angle and frequency. Here, the impinging SWs are excited on the left-sided or right-sided plasmonic metal to travel through the PB metasurface (adopted in Figure 3 of the manuscript) along +x (A, C, E) or -x (B, D, F) direction

# F. Far-field emissions of SWs with PB metasurfaces of different phase gradients

**
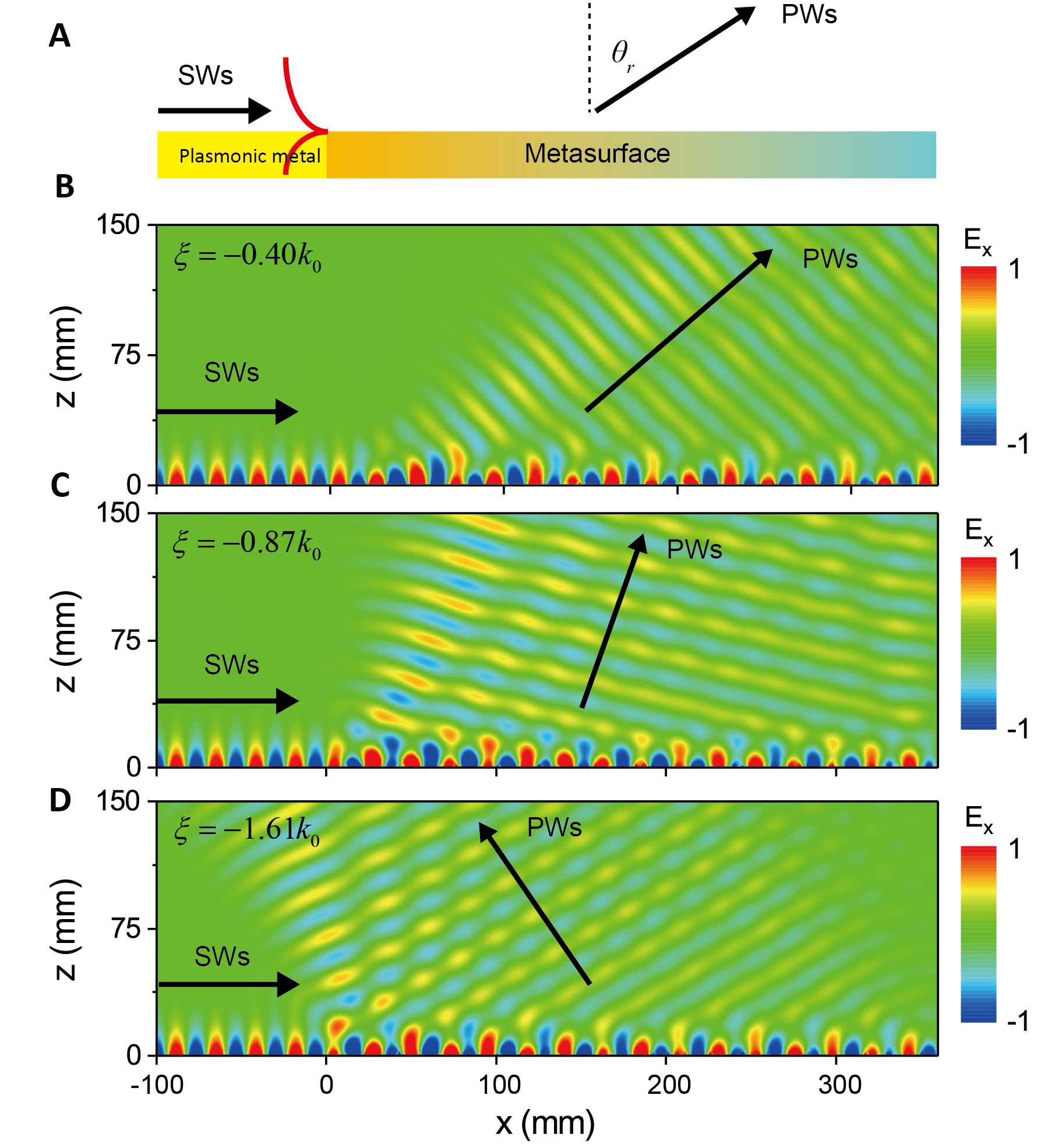
**

FIG. S6: (A) Schematic of the plasmonic meal (left) and PB metasurface (right) with a linear phase profile for SW-PW radiation. (B-D) Simulated E_x_ field distribution of SW-PW unidirectional emission by PB metasurfaces of different phase gradients, i.e., $\xi=-0.40k_{0},-0.87k_{0}\text{,}-1.61k_{0}$(corresponding to Figure 4B). It is noted that the radiation angles are $45^{\circ},14^{\circ},-30^{\circ}$in three systems, respectively. Here, the frequency is 12GHz.

# G. Far-field radiations of SWs with PB metasurfaces of inhomogeneous PCRs


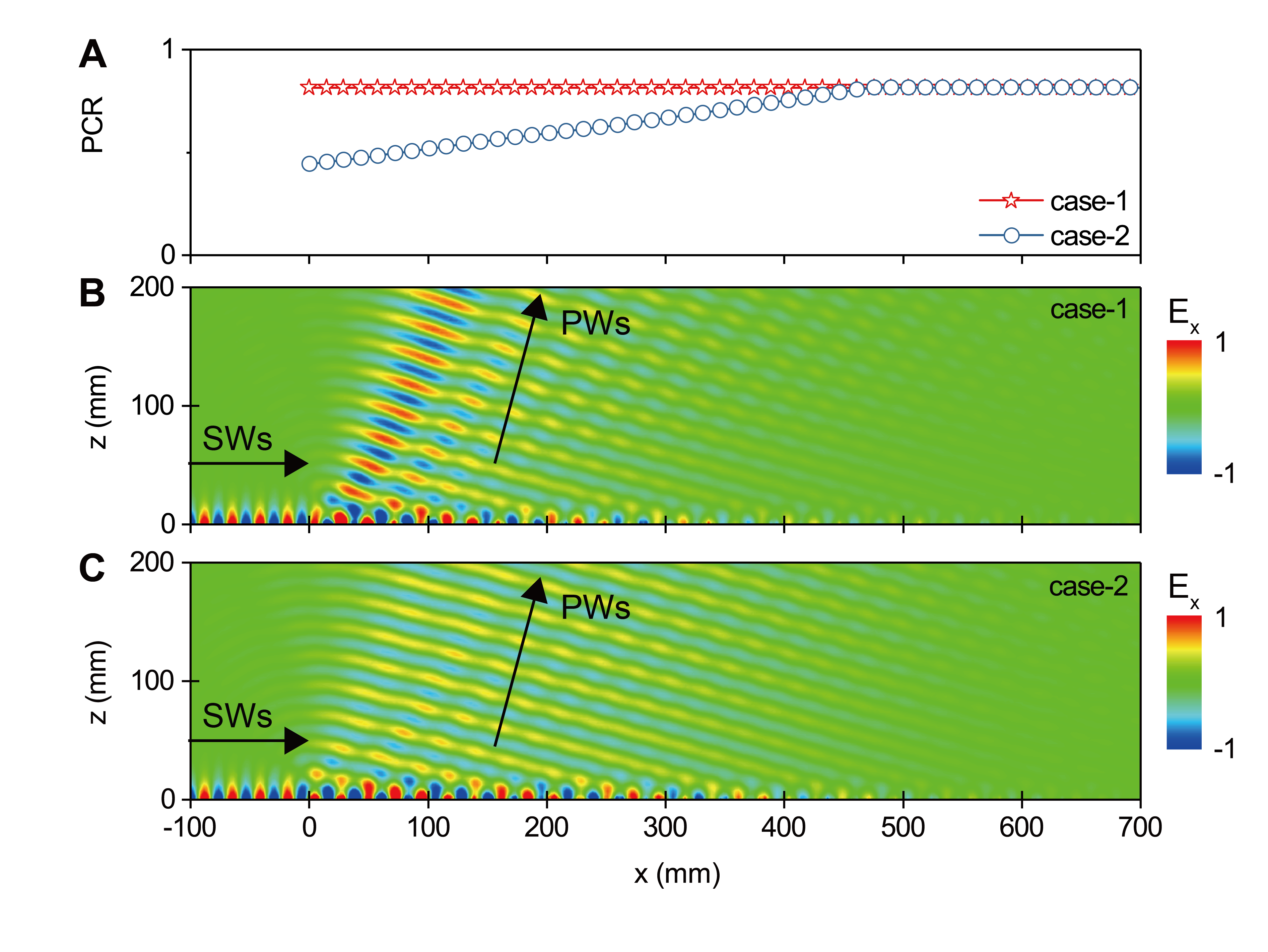


FIG. S7 Simulated E_x_ field distributions to illustrate the unidirectional emission of SWs in two different PB metasurfaces, where the adopted PB meta-atoms possess (B) homogeneous (PCR=0.8, corresponding to Figure 5E in the main text) or (C) inhomogeneous PCR distribution as shown in (A). Here, the frequency is 12GHz.

**
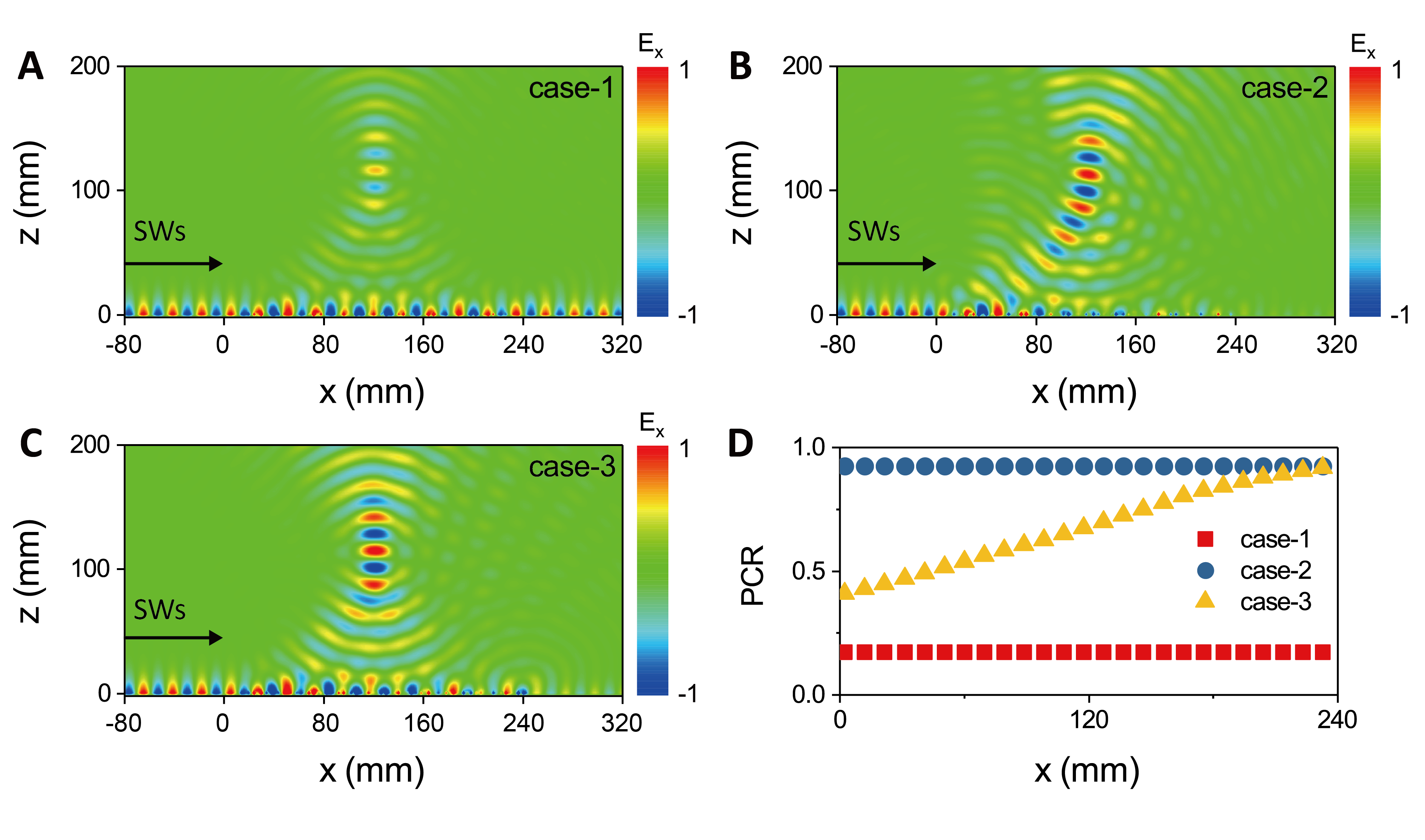
**

FIG. S8: (A)-(C) Simulated E_x_ field distributions to illustrate the line focusing of SWs in three different PB metasurfaces, where the adopted PB meta-atoms possess (A, B) homogeneous or (C) inhomogeneous PCR distribution along x direction as shown in D. Here, the frequency is 12GHz.
